# An Exploration of the Social Brain Hypothesis in Insects

**DOI:** 10.3389/fphys.2012.00442

**Published:** 2012-11-27

**Authors:** Mathieu Lihoreau, Tanya Latty, Lars Chittka

**Affiliations:** ^1^School of Biological Sciences, The University of SydneySydney, NSW, Australia; ^2^The Charles Perkins Centre, The University of SydneySydney, NSW, Australia; ^3^Psychology Division, School of Biological and Chemical Sciences, Queen Mary University of LondonLondon, UK

**Keywords:** cognition, insects, mushroom bodies, sociality, social brain hypothesis

## Abstract

The “social brain hypothesis” posits that the cognitive demands of sociality have driven the evolution of substantially enlarged brains in primates and some other mammals. Whether such reasoning can apply to all social animals is an open question. Here we examine the evolutionary relationships between sociality, cognition, and brain size in insects, a taxonomic group characterized by an extreme sophistication of social behaviors and relatively simple nervous systems. We discuss the application of the social brain hypothesis in this group, based on comparative studies of brain volumes across species exhibiting various levels of social complexity. We illustrate how some of the major behavioral innovations of social insects may in fact require little information-processing and minor adjustments of neural circuitry, thus potentially selecting for more specialized rather than bigger brains. We argue that future work aiming to understand how animal behavior, cognition, and brains are shaped by the environment (including social interactions) should focus on brain functions and identify neural circuitry correlates of social tasks, not only brain sizes.

As intelligence dominates on instinct, the mushroom bodies and the antennal lobes become considerably larger relative to the overall brain volume, as we see when comparing cockchafers to locusts, ichneumons, carpenter bees, solitary bees, and finally social bees, where the mushroom bodies represent 1/5th of the brain and 1/940th of the body; whereas in cockchafers, they represent less than 1/33,000th of the body.

Translated from Dujardin (1850, p. 202)

## The Social Brain Hypothesis

There have been suggestions that the cognitive challenges of managing social relationships in groups of increasing size have driven the evolution of large brains, with more neurons and enhanced information-processing capabilities, and that this trend is at the root of human intelligence (Jolly, 1966; Humphrey, 1976; Byrne and Whiten, 1988; Dunbar, 1998). This “social brain” hypothesis has received some support from correlations between measures of brain size and proxies for social complexity in various mammals (Dunbar and Shultz, 2007; Pérez-Barberia et al., 2007). In anthropoid primates, for instance, the ratio of neocortex to total brain size increases with species’ typical group size (Dunbar, 1998). At the individual level, the volumes of the amygdala (Sallet et al., 2011) and the prefrontal cortex (Powell et al., 2012) also appear to vary with the size of the social network. However, despite these observations, many anthropologists and neuroscientists are concerned that this hypothesis may be too simplistic to account for the complex evolution of animal brains (Balter, 2012). Some authors question whether many of the forms of social cognition found in group-living animals have a unique social component (as solitary animals also acquire information from other individuals; Heyes, 2012), or whether differences in brain size result from changes in cognitive capacity unrelated to sociality (Barton, 2004; Farris and Roberts, 2005; Healy and Rowe, 2007; Finarelli and Flynn, 2009).

**Figure 1:**
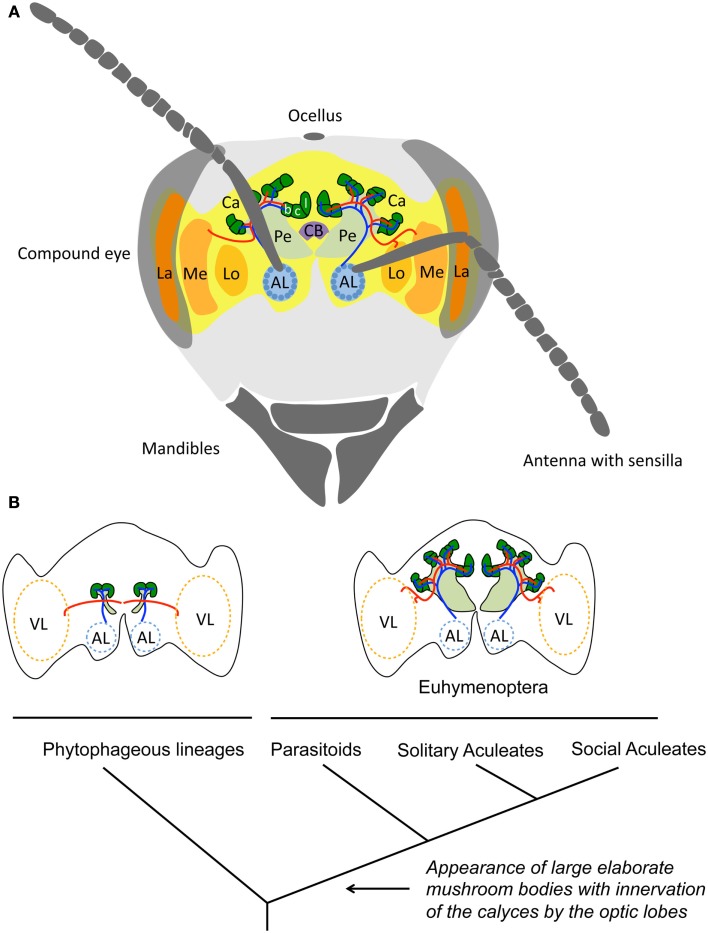
**Comparing insect brain sizes**. **(A)** Schematic drawing of a honeybee worker brain (*Apis mellifera*). The visual lobes (VL) composed of the lamina (La), the medulla (Me), and the lobula (Lo), receive the sensory inputs from the compound eyes. The antennal lobes (AL) receive the inputs from the olfactory sensory neurons of the antennae in spherical subunits (glomeruli) that represent particular aspects of an odor. The mushroom bodies are central structures that process multimodal information and participate in learning and memory. These structures have a characteristic morphology consisting of a pedunculus (Pe) and two calyces (Ca) compartmentalized into the lip (l), which receives olfactory input from the antennal lobes (blue neural network), the collar (c), which receives input from the visual lobes (red neural network), and the basal ring (b), which receives combined input from the antennal and visual lobes. The central body (CB) has connections to all major parts of the brain and is involved in leg coordination and motor control. Image modified from Menzel and Giurfa (2001). **(B)** Schematic representation of the morphology of the mushroom bodies of Hymenoptera, mapped on a simplified phylogeny, based on Farris and Schulmeister (2011). The mushroom bodies of phytophageous lineages are small, the calyces lack subcompartmentalization and do not receive visual inputs from the optic lobes. Large mushroom bodies with lip, collar and basal ring subcompartments in the calyx, and visual inputs arose concurrent with the acquisition of a parasitoid mode of life at the base of the Euhymenopteran, ca 90My prior to the evolution of social aculeates, such as honeybees.

The link between sociality and brain area size was originally proposed for social insects long before the idea emerged in primate research, by Dujardin (1850), who observed that the mushroom bodies (structures of the insect brain involved in learning and memory; Figure 1A) are substantially enlarged in honeybees (see above). Dujardin suggested that these brain areas were the seat of insect “intelligence,” and since then, a relationship between large mushroom bodies, advanced cognition, and sociality has been assumed, although rarely explicitly tested (Strausfeld et al., 1998). Insects provide a unique opportunity to further explore these questions since they exhibit an unparalleled diversity of social forms – from temporary aggregations (Costa, 2006) to permanent colonies containing millions of individuals working together as a “superorganism” (Hölldobler and Wilson, 2009) – and have evolved an impressive range of cognitive skills despite their miniature nervous systems (Chittka and Niven, 2009).

Here we explore the evolutionary relationships between sociality, cognition, and brain size in insects. We review comparative studies of brain anatomy testing predictions of the social brain hypothesis. We illustrate how some major behavioral innovations of social insects may in fact require relatively simple and computationally inexpensive forms of cognition. We then argue that the cognitive demands of sociality should be investigated in terms of the sensory information being used, the computational challenges for defined social-cognitive tasks, and the neural networks they require, not just brain sizes.

## Comparative Analyses of Insect Brain Size

Although some insects exhibit extreme social sophistication that may have few equivalents in vertebrates, many of their social interactions are governed by simple behavioral routines. In the most integrated societies, individuals sometimes specialize in a single task (reproduction, brood care, defense, or foraging) and may require fewer cognitive capabilities than in simpler groups where individuals perform multiple tasks. Therefore, rather than a single positive correlation between brain size and group size, Gronenberg and Riveros (2009) suggest that the volume of insect brains (or specific brain components) should increase with ascending degrees of sociality in small “individualized” societies. Brain volume, however, should decrease in the larger and decentralized “class-based” societies, characterized by division of labor. We review recent studies exploring these predictions below.

### Interspecific variation

If sociality involves processing and storing more information, certain brain structures might be enlarged in social insects compared to solitary ones. Early observations suggest that this is the case in Hymenoptera (Dujardin, 1850; von Alten, 1910; Howse, 1974). However, Farris and Schulmeister (2011) recently challenged these observations by comparing the mushroom bodies of 22 hymenopteran species. Their study demonstrate that large mushroom bodies with elaborated calyces, receiving inputs from visual and olfactory neuropils, are equally common in solitary, presocial, and eusocial Hymenoptera. In fact, the study suggests that large mushroom bodies were acquired 90Myr before the evolution of sociality in the group and coincided with the transition from phytophagy to parasitoidism in solitary wasps (Figure 1B). Presumably, the novel challenges of central place foraging, finding, and overwhelming prey to provision larvae, and especially the need to develop spatial memories for their nesting sites, placed much higher cognitive demands on these parasitoids than their vagabond herbivorous ancestors. A similar approach in non-hymenopteran insects confirms that the evolution of large mushroom bodies is primarily associated with complex foraging behavior rather than with sociality. Examples include generalist scarab beetles that must discriminate among many potential resources to balance their diet in heterogeneous nutritional environments (Farris and Roberts, 2005), or pollinating butterflies that exploit flowers using habitual foraging routes (Sivinski, 1989). Indeed, many of the most impressive cognitive feats in insects have been identified in the context of individual foraging and are unrelated to sociality (e.g., attention-like processes, Spaethe et al., 2006; interval timing, Boisvert and Sherry, 2006; numerosity, Chittka and Geiger, 1995; rule learning, Giurfa et al., 2001; and route optimization Lihoreau et al., 2012). Recent studies have begun to focus on other brain components, and suggest that the size of antennal lobes (Figure 1A) varies with social organization in wasps (Molina et al., 2009) and ants (Riveros et al., 2012). It will be important to explore which differences in internal structure mediate these changes; for example, a larger volume of antennal lobe glomeruli might indicate higher sensitivity to certain odorants, whereas a larger number of glomeruli might mediate a sensitivity to a higher diversity of chemicals such as pheromones.

### Intraspecific variation

Some adult insects exhibit dramatic structural plasticity in their brains in the form of dendritic outgrowth (Withers et al., 1993) or neurogenesis (Ott and Rogers, 2010), which also provides a potential test of the social brain hypothesis. If sociality requires added circuitry, brain size should increase in individuals that change from being solitary to social at different stages of their life cycle. In the facultatively social bee *Megalopta genalis*, where cooperatively breeding females establish a dominance hierarchy to determine their contribution to reproduction, the mushroom bodies of dominant reproductives are much larger than those of subordinate workers and solitary reproductives (Smith et al., 2010). Similar enlargement of the mushroom bodies of reproductives is found in multiple obligatorily social wasp species (Molina and O’Donnell, 2007, 2008; O’Donnell et al., 2011), suggesting that the demands of competing over reproduction or maintaining dominance (and thus remembering others’ identity, assessing their status, and displaying aggressive behaviors) promote mushroom body growth (but see Ehmer et al., 2001). Thus while in this case there is a link between social behavior and mushroom body volume, it does not equally affect all members of the colony.

Another striking example of neuroplasticity in the adult brain is the continuous axonal pruning and dendritic outgrowth in the calyces of the mushroom bodies of social Hymenoptera. In honeybee workers (*Apis mellifera*), the mushroom bodies enlarge before the transition to becoming foragers, while individuals still remain inside the hive and engage in brood care duties (Fahrbach et al., 1998). This “experience-expectant” plasticity prepares bees to store memories related to outdoor navigation, which involves exploring multiple foraging options, learning flower locations, their sensory signals, and handling procedures. Actual foraging experience also triggers a further “experience-dependent” increase in mushroom body size (Withers et al., 1993). This plasticity is not specific to honeybees but has been observed in ants (Gronenberg et al., 1996; Kuhn-Buhlmann and Wehner, 2006), wasps (O’Donnell et al., 2004), and solitary bees (Whiters et al., 2007), thus emphasizing the tight relationship between foraging and the development of mushroom bodies in these insects.

## What are the Cognitive Challenges Imposed by Sociality?

An alternative approach to examining the evolution of sociality and cognition is to ask what the computational nature of a social task is, and what neural circuitry might actually be required to accomplish this task. Answering these questions will clarify the extent to which the acquisition of a social trait requires greater cognitive loads and added neural circuitry. We discuss below some major behavioral innovations of social insects.

### Nestmate recognition in ants

In humans, societies are held together by the ability of individuals to recognize generalized features indicating group membership. This allows them to identify society members without needing to memorize the identity of every single individual in the group. Social insects, likewise, often recognize members of their own colony, allowing them to defend resources and brood against competitors. It is important to keep in mind that with the advent of the generalized “labels” defining members of a society (be they tribe-specific clothing in humans or chemical profiles in insects) the size of a society essentially becomes infinite. Identifying group members does not require any added cognitive demand; individuals need only remember a set of defining features. In ants, nestmate recognition is mediated by colony-specific chemosensory cues on the cuticle of colony-members (van Zweden and D’Ettorre, 2010). Ants recognize “friends” and “foes” by comparing the odor carried by encountered individuals to an internal representation of their own colony odor, which requires fine discrimination of multicomponent variable cues (Guerrieri et al., 2009). Neuroethological studies suggest that such recognition can be achieved through simple cognitive operations that do not involve information-processing in the higher integration centers of the brain or the acquisition of a long-term memory (Bos and D’Ettorre, 2012). For instance, electrophysiological recordings of the antennae of *Camponotus japonicus* have shown that cuticular hydrocarbon-sensitive chemosensilla are less responsive to non-nestmate odor upon prolonged exposure, suggesting that receptors adapt to the chemical environment and act as a “sensory filter” mediating recognition (Ozaki et al., 2005). Decreased responses to familiar odors could also result from non-associative learning (such as habituation) at the level of the antennal lobe, as suggested by the activity patterns of antennal lobes subsequent to exposure with colony odors in *C. floridanus* (Brandstaetter et al., 2011) and the absence of information transfer between brain hemispheres (through the mushroom bodies) during recognition in *C. aethiops* (Stroeymeyt et al., 2010). Although it is still not clear precisely where colony odors are stored and processed in the ant brain, the template is decentralized in the olfactory system and recognition is achieved through simple processes such as sensory adaptation or habituation.

### Individual recognition in wasps

**Figure 2:**
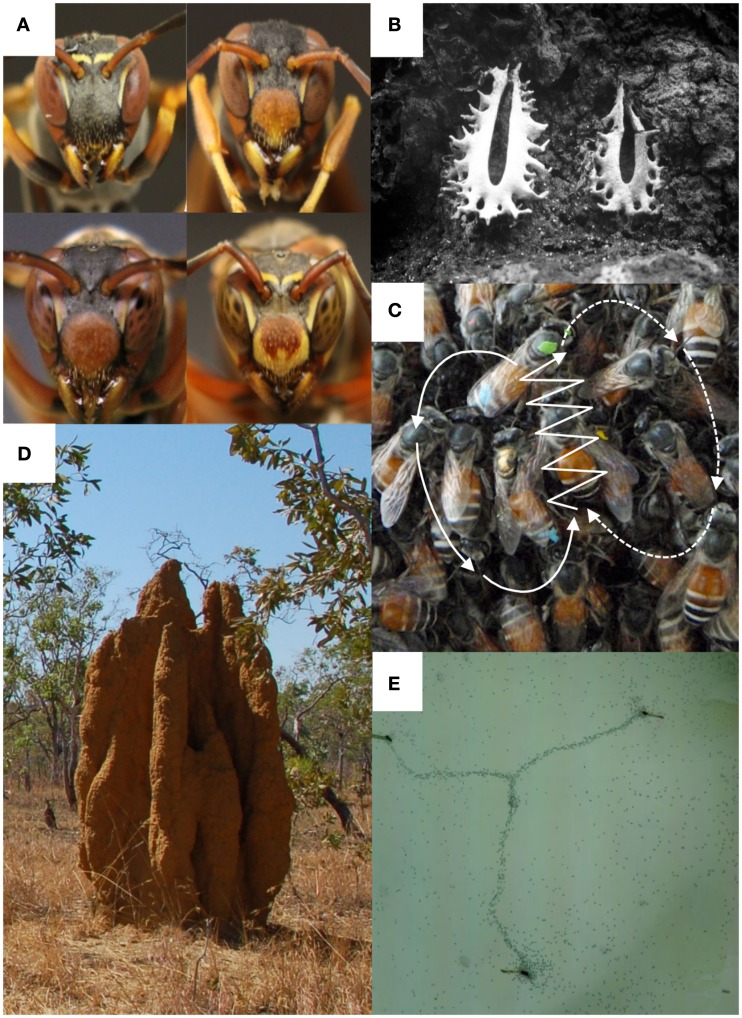
**Examples of socio-cognitive tasks by social insects that might involve computationally inexpensive cognition and minor adjustment of neural circuitry**. **(A)** In small colonies of the wasps *Polistes fuscatus*, cooperatively breeding females learn the identity of every other females based on their conspicuous facial markings. Face recognition helps stabilize social interactions and reducing aggressions (photos M. J. Sheehan). **(B)** Each colony of the Brazilian stingless bee *Partamona pearsoni* marks its nest entrance with a visually unique structure, which defines the colony’s identity. Using colony-specific identifiers, a society can theoretically become infinitely large without increasing the cognitive demands on its members (photo J. M. F. Camargo). **(C)** Honeybees, such as *Apis florea*, communicate food and potential nest locations to their nestmates using a “dance” language. Upon its return to the nest, a successful forager performs a figure-of-eight-shaped circuit (white arrows) conveying information about the distance (duration of the waggle phase) and the direction (body orientation relative to gravity) of the target resource (photo J. Makinson). **(D)** The Australian termites *Nasutitermes triodiae* build complex “cathedral” nests several meters high containing multiple chambers. Walls and pillars arise through the action of many termites, each depositing soil pellets at sites scented with an attractive cement pheromone, which coordinates the accumulation of building materials without any individual having a global knowledge of the construction process (photo S. Scheurer). **(E)** Argentine ants *Linepithema humile* develop pheromone transportation networks to connect the multiple nests of the colony. Collectively, ants establish near optimal networks using pheromone trails as an “externalized memory” (photo T. Latty).

In some small colonies of primitively social ants (D’Ettorre and Heinze, 2005) and wasps (Tibbetts, 2002), females establish a dominance hierarchy and develop a long-term memory of colony-members’ identity, which helps stabilize social interactions. In *Polistes* wasps, individual recognition is mediated through conspicuous facial markings (Sheehan and Tibbetts, 2008; Figure 2A). These wasps are naturally specialized “experts” in face recognition since they are less efficient at recognizing arbitrary patterns, or face pictures that lack antennae or contain scrambled features (Sheehan and Tibbetts, 2011). Comparisons of brains from wasp species that learn faces and closely related species that lack face recognition revealed no discernable difference in the size of the visual neuropils (Gronenberg et al., 2008). Presumably, minor modifications of the pre-existing neural circuitry used for recognition of landmarks or prey in the common ancestors of these wasps have sufficed to evolve individual recognition. Artificial neural networks confirm that basic circuitry for face recognition requires only a few hundred neurons (Aitkenhead and McDonald, 2003) and could thus easily be accommodated in the insect brain. Similar specializations of the visual system could potentially underpin a variety of social recognition processes, as for example the ability of some stingless bees to recognize nest identities using visual marks (Chittka et al., 1997; Figure 2B).

### The dance language of honeybees

The symbolic “dance language” of honeybees is perhaps the most impressive form of communication found in insects (von Frisch, 1967). Upon its return to the nest, a bee that has located a new food resource performs the “waggle dance,” a figure-of-eight-shaped circuit conveying information about the distance and the direction of the resource to its nestmates (Figure 2C). In an even more impressive social use of the dance language, bees indicate suitable nest site locations to a swarm in search of a new home. The swarm builds a consensus from multiple “opinions” expressed by scouts with different information, to finally agree on a single destination to which the swarm relocates (Seeley, 2010). At least five sensory systems (motion sensitivity, sun compass, gravity, mechanosensitivity, and acoustic perception) are required to acquire, transmit, and decode the information of the dance language. Therefore, one might expect this unique behavioral innovation to have easily detectable correlates in terms of neuroanatomy. However, examination of the neural pathways mediating these sensory systems have revealed no “dance specific” projections in the brain of honeybee workers (*A. mellifera*) when compared with queens, drones, and workers of species that lack dance communication (Brockmann and Robinson, 2007). This suggests that small tweakings of existing circuitry, such as novel connections between sensory pathways, central circuits, and motor output may have sufficed to produce this behavior. It is easy to imagine, for example, how circuitry in place for forward walking locomotion can be supplemented by a few control neurons to recruit the leg muscles in the order required to generate a figure-eight pattern.

### Trail pheromone networks in ants

Many of the collective behaviors of insects, such as consensus building during nest site selection by honeybees (Seeley, 2010) and ants (Sasaki and Pratt, 2012), foraging decisions by cockroaches (Lihoreau et al., 2010) or nest construction by termites (Bonabeau et al., 1998; Figure 2D), emerge through self-organization, based on local interactions between partially informed individuals (Jeanson et al., 2012). Through communication, insects can overcome individual limitations in acquiring or processing information, allowing groups to make faster and more accurate decisions than they might as individuals (Couzin, 2009). Seeley (2010) makes a convincing case that workers in a honeybee swarm can essentially be viewed as sensory units of a “collective brain”. The mechanisms behind this “swarm intelligence” have been particularly well described in the context of path optimization in ants (Goss et al., 1989). Argentine ants (*Linepithema humile*), for instance, develop networks connecting the multiple nests of their colonies (Aron et al., 1990). Trails arise through the action of many ants, each depositing droplets of an attractive pheromone at regular intervals as they explore their environment. The initial network is complex, containing many redundant connections. But pheromones evaporate, so longer, more circuitous routes (which take longer to traverse and so get fewer passes) evaporate first, while shorter, more direct routes are reinforced more often and last longer. The end result of this positive feedback loop is a network that connects nests via the shortest path (Latty et al., 2011; Figure 2E). In such decentralized systems, pheromone trails serve as an “externalized memory” freeing individual ants from the need to store location information in their brains, whilst allowing colonies to reach network optimization performances not accessible to isolated individuals.

## Concluding Remarks

There is no question that insect cognition is shaped by ecology, including the social environment. However, so far, studies aiming to correlate brain volumes to social complexity have failed to identify clear correlations between sociality, cognition, and the size of particular neuropils. While many of the social behavior routines of social insects are innate, they must have neural correlates, and the adjustments in neural wiring needed to evolve such routines might be too minor to be readily detectable in terms of gross neuroanatomy. If we wish to elucidate how sociality shapes cognition, it is essential to determine the actual cognitive load of a defined behavior and identify how many neurons, connections, and sequential stages of information-processing are required to perform that behavior.

Insects, with their relatively small nervous systems, hold considerable potential to explore the neural underpinnings of sociality. Neuron-to-neuron connectivities and their role in learning and memory can increasingly be investigated from both empirical and modeling perspectives. Recently, 16% of the ∼100,000 neurons of the *Drosophila* brain have been mapped (Chiang et al., 2011), illustrating just how far we have progressed in understanding the full circuitry of small brains. Calcium imaging (Joerges et al., 1997) and multi-electrode recording (Bender et al., 2010) techniques now enable investigations of activity patterns of neurons at the individual cell and circuitry levels, with the potential to assess the function of neural circuits in the learning and processing of social cues by individuals. Artificial neural networks are also extremely useful in developing crisp hypotheses and identifying circuitries that might enable various forms of social cognition, such a imitation (Laland and Bateson, 2001), face recognition (Aitkenhead and McDonald, 2003), or phenomena that might be interpreted as theory of mind (van der Vaart et al., 2012). For example, we lack clear predictions about how many changes in neural circuitry might be necessary to re-deploy a visual system evolved for identifying visual landmarks or prey for the purposes of face recognition. Once such a system is in place to identify, say, 10 faces, how much more would it take to identify a hundred? Would this require copying the same circuitry multiple times, as implied by the original version of the social brain hypothesis, which held that relative brain size increases with the number of individuals in the group (Dunbar, 1998)? Or would there be more efficient ways to increase memory storage, possibly involving neuronal re-use (Anderson, 2010)? What added circuitry might be required to mediate a qualitative advance related to face recognition, such as recognizing them from various vantage points? Although artificial neural networks are perhaps unlikely to be similar to those implemented in real brains, they might be useful in identifying the minimum number of neurons necessary to perform a given task, and in developing hypotheses for the number of evolutionary changes needed to generate a novel cognitive capacity. Artificial neural networks can therefore guide the search for the real neural implementations of specific tasks. A “bottom-up” approach from neuroscience to comparative cognition and sociobiology (Chittka et al., 2012), will help refine current hypotheses of the evolution of socio-cognitive abilities, not only in small-brained insects, but more generally in all social animals.

## Conflict of Interest Statement

The authors declare that the research was conducted in the absence of any commercial or financial relationships that could be construed as a potential conflict of interest.
